# Flow Cytometry Approach to Quantify the Viability of Milk Somatic Cell Counts after Various Physico-Chemical Treatments

**DOI:** 10.1371/journal.pone.0146071

**Published:** 2015-12-30

**Authors:** Na Li, Romain Richoux, Marie-Hélène Perruchot, Marion Boutinaud, Jean-François Mayol, Valérie Gagnaire

**Affiliations:** 1 INRA, UMR 1253, Science et Technologie du Lait et de l’Œuf, 35042 Rennes, France; 2 Agrocampus Ouest, UMR 1253, Science et Technologie du Lait et de l’Œuf, 35042 Rennes, France; 3 Actalia, BP 50915, 35009 Rennes, France; 4 INRA, UMR 1348, Physiologie, Environnement et Génétique pour l’animal et les systèmes d’élevage, 35590 Saint Gilles, France; 5 Agrocampus Ouest, UMR 1348, Physiologie, Environnement et Génétique pour l’animal et les systèmes d’élevage, 35000, Rennes, France; 6 R&D TranscureBioservices, 74160 Archamps, France; Université de Technologie de Compiègne, FRANCE

## Abstract

Flow cytometry has been used as a routine method to count somatic cells in milk, and to ascertain udder health and milk quality. However, few studies investigate the viability of somatic cells and even fewer at a subpopulation level to follow up how the cells can resist to various stresses that can be encountered during technological processes. To address this issue, a flow cytometry approach was used to simultaneously identify cell types of bovine milk using cell-specific antibodies and to measure the cell viability among the identified subpopulations by using a live/dead cell viability kit. Confirmation of the cell viability was performed by using conventional microscopy. Different physico-chemical treatments were carried out on standardized cell samples, such as heat treatment, various centrifugation rates and storage in milk or in PBS pH 7.4 for three days. Cytometry gating strategy was developed by using blood cell samples stored at 4°C in PBS and milk cell samples heat-treated at 80°C for 30 min as a control for the maximum (95.9%) and minimum (0.7%) values of cell viability respectively. Cell viability in the initial samples was 39.5% for all cells and varied for each cell population from 26.7% for PMNs, to 32.6% for macrophages, and 58.3% for lymphocytes. Regarding the physico-chemical treatments applied, somatic cells did not sustain heat treatment at 60°C and 80°C in contrast to changes in centrifugation rates, for which only the higher level, i.e. 5000×*g* led to a cell viability decrease, down to 9.4%, but no significant changes within the cell subpopulation distribution were observed. Finally, the somatic cells were better preserved in milk after 72h storage, in particular PMNs, that maintained a viability of 34.0 ± 2.9% compared to 4.9±1.9% in PBS, while there was almost no changes for macrophages (41.7 ± 5.7% in milk *vs* 31.2 ± 2.4% in PBS) and lymphocytes (25.3 ± 3.0% in milk *vs* 11.4 ± 3.1% in PBS). This study provides a new array to better understand milk cell biology and to establish the relationship between the cell viability and the release of their endogenous enzymes in dairy matrix.

## Introduction

Milk naturally contains somatic cells besides the well-known biochemical components, i.e. water, lactose, protein, fat, minerals… These milk somatic cells are made up of four main cell types: macrophages, polymorphonuclear neutrophils (PMNs) and lymphocytes that exist initially in blood and epithelial cells in the mammary glands. The immune cells are involved in the defense of mammary glands, especially PMNs [1] and the global somatic cell count is used as an undisputed criterion of udder health and milk quality [2,3].

Somatic cells are important sources of various enzymes depending on the types of cells present, in particular proteases and lipases, that can be released during milk technological processes and further impact the final characteristics of milk products. Whether the cells can resist or not to various stresses encountered during technological processes are still under question.

Flow cytometry is a favored method used to have information on the physiological status of somatic cells after milking. Indeed, this accurate and reproducible method is routinely used to evaluate the total number of somatic cells present in milk of different species [4,5]. Thanks to the labeling with specific antibodies, already developed, macrophages, PMN and subtypes of lymphocytes are monitored in milk [3–6]. Moreover, some studies characterized lymphocytes by Forward Scatter (FSC) and Side Scatter (SSC) dot plots [7]. To quantify the cell viability, the exclusion markers i.e. propidium iodide, 7-Aminoactinomycin D, acridine orange or their combination are usually used to distinguish the viable and dead cells.

However, flow cytometry has rarely been used to measure the global viability of the somatic cells and *a fortiori* for each cell type except on a single subpopulation, the PMNs in milk [4,5,8], in human blood, and in horse synovial fluid [9,10]. Recent studies demonstrate that each subpopulation of milk somatic cells is able to provide its own profiles of endogenous enzymes in terms of enzyme type, quantity, specificity and activity and give a fingerprint of potential activities that could be released in milk [11] and in turn could affect milk quality as well as the manufacture and quality of dairy products [12]. We aimed to develop a flow cytometry method to measure the cell viability with a live/dead kit of total somatic cell counts and of differentiate somatic cells in milk. As cells could release their intracellular content when the membrane integrity is lost, the resistance of milk somatic cells after milking was tested under various physico-chemical conditions.

## Materials and Methods

**Fig 1:**
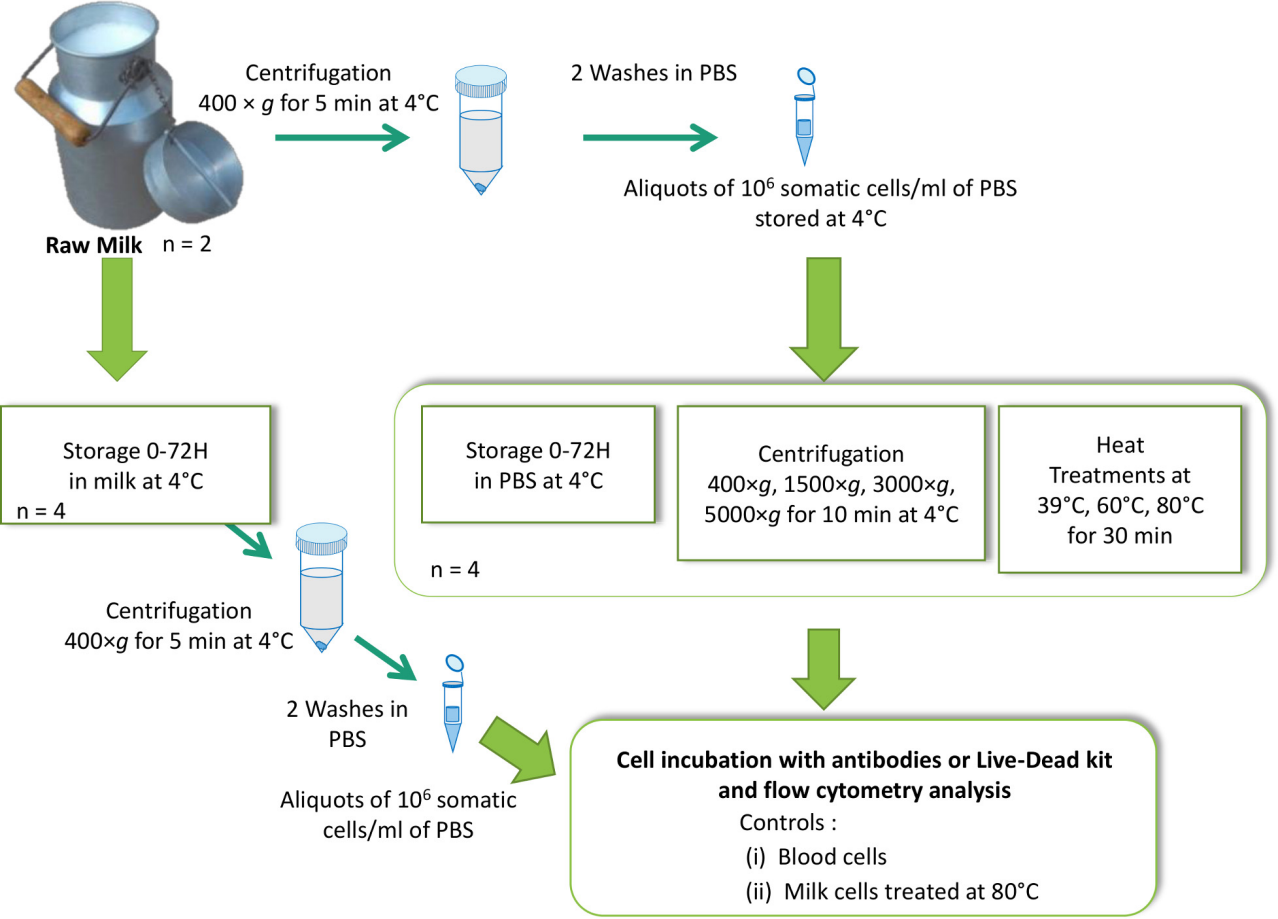
Summary of the experimental design of the somatic cell preparation and the various treatments applied, i.e. storage in milk or PBS for 72 h, variation of the centrifugation rates and heat treatment.

The whole experimental design is presented in Fig 1, and the corresponding steps are developed in the different sections below.

### Milk Somatic Cell Isolation

Raw milk was obtained from a bulk of 30 clinically healthy Jersey cows from a farm that commercializes milk according to the European directive 92/46/CEE with bacterial contamination less than 10^5^ ufc/ml and somatic cells less than 400 000 cells/mL. Actually the milk mesophilic aerophilic flora was estimated between 1 and 8 10^3^ colony forming unit /mL by using standard plate count agar at 30°C for 3 days and the average amount of somatic cells was 273 000 cells/mL of milk. Somatic cell enumeration in milk was performed by an independent dairy laboratory (Lillab, Chateaugiron, France) using the reference method ISO1336-2/IDF148-22006 [13]. Milk was transported in sterile tubes under refrigerated conditions for further analysis. Raw bulk milk (~25 l per assay, n = 2 independent assays) was firstly centrifuged at 400×*g* during 10 min to collect the cells and to eliminate fat globules. The rest of milk volume was kept at 4°C for testing viability during storage period (cf “physico-chemical treatments applied to collected somatic cells from milk” of the material and method section). The cell pellet obtained was washed twice at 400×*g* 10 min with cold phosphate buffer saline (PBS) pH 7.4 and stored in PBS at 4°C. The milk cell suspensions were filtrated through a 40 μm cell strainer to eliminate aggregates and to obtain single cell suspensions (BD Bioscience, Le Pont de Claix, France), and then counted on Malassez counting slide (Dutscher, Brumath, France) diluted with cold PBS to obtain a standardized concentration of 10^6^ cells/mL. Milk cell suspensions were distributed as 200 μL aliquots for further cell analyses.

### Blood Somatic Cell Isolation

Peripheral blood samples were taken from the cows used for milking, in accordance with the general directive on animal care used in the European Community [14]. All the procedures applied to animals (milking and blood samples) were approved by the Animal Care Committee of the French Ministry of Agriculture, in accordance with French regulations (Decree No. 2001–464, May 26, 2001). The cows were housed at the INRA Méjusseaume experimental dairy farm, UMR1348 IEPL (Le Rheu, France) with C35-275-23 as the agreement number for animal research (06 october 2010). One single blood sample was obtained from the tail vein from a cow physically restrained. The jugular vein is the most common site for blood collection in cows and good physical restraint is an acceptable technics that does not affect the animal's integrity. All efforts were made to minimize suffering.

We confirm that the field studies did not involve endangered or protected species.

Peripheral blood from the jugular vein was collected by venipuncture in 5 mL ethylene diamine tetra acetic acid (EDTA) tubes. Erythrocytes were lysed through brief hypotonic shock by adding 4.5 mL of cold Milli-Q water/ml of blood for 15 sec. Erythrocyte cell lysis was stopped using 500 μL cold 10×PBS [15]. The blood somatic cells were centrifuged, filtered under the same conditions as described for milk somatic cell isolation and standardized to 10^6^ cells/mL in PBS and stored as 200 μL aliquots for further cell analyses.

### Flow cytometry analyses

All measurements were performed on the flow cytometer MACS Quant (Miltenyi Biotec SAS, Paris, France) equipped with three lasers, red laser at 633 nm, blue at 488 nm and violet at 405 nm. All cell samples for flow cytometry analyses were layered into 12 × 75 mm polypropylene round-bottom tubes (BD Biosciences). The samples were run at medium speed, and 10 000 events were collected for each cytometry sample. Compensation beads (BD Biosciences) were used according to the manufacturer’s procedure to compensate for channel cross-talk. All data were acquired and analyzed by means of MACS Quantify analyzer software (Miltenyi Biotec SAS).

In our study, a mixed sample of milk and blood cell suspension (volume 1:1 ratio) was used to validate the analytical scatter pattern. The individual fresh blood and the milk cell suspensions after 30 min at 80°C were used to provide the maximum and the minimum cell viability values, respectively and were prepared as shown below. The milk cell suspensions stored in PBS at 4°C were considered as the reference sample compared to the other physico-chemical treatments applied to the cells.

#### Incubation with specific antibodies for monitoring cell distribution

Cell suspensions were immediately incubated with the antibodies and to ensure cell viability and avoid fluorophore photobleaching, all incubation and centrifugation steps described below were carried out in the dark at 4°C. The primary and secondary antibodies shown in Table 1 were chosen according to the literature data, their market availability and their compatibility with the optical configuration of the cytometer MACS Quant. The labeling fluorophore relative fluorescence emission intensity and the extent of labeling were also considered for the selection of secondary double antibodies.

**Table 1:** List of antibodies, fluorescence markers and isotype control for differentiation and labeling of bovine somatic cells applied on flow cytometry.

	1^st^ mAb (a-bovine) mouse		2^nd^ mAb (a-mouse) mouse		Isotype	Isotype Control
Target cells	Name/Clone	Reference	Fluorescence	Reference		Reference
All cells	CD45-CACTB51A	Bov2039^1^	PerCP	F0131 ^2^	IgG2a	IC003C ^2^
PMNs	CH138A	Bov2067^1^	FITC	553408 ^3^	IgM	555583 ^3^
Monocytes/Macrophages	CD14-CAM36A	Bov2027^1^	APC	560089 ^3^	IgG1	555751 ^3^

^1^W.S.U. Monoclonal antibody center, veterinary microbiology and pathology, Pullman WA, United States.

^2^R&D systems, Minneapolis, United States.

^3^BD Biosciences, Becton Dickinson, France.

To label all somatic cells of milk except epithelial cells, i.e. immune cells, and cow peripheral blood, the anti-bovine CD45 (IgG2a) mouse primary monoclonal antibodies (mAb) were chosen (Bov 2039, VMRD Inc., Pullman WA). Primary mAb against the bovine CD14 (IgG1) protein was used to detect specifically macrophages (Bov 2027, VMRD Inc.). Primary mAb against the bovine CH138A (IgM) protein was used (Bov 2067, VMRD Inc.) to detect granulocytic cells, which encompass neutrophils eosinophils PMNs according to the manufacturer’s specifications and some related studies as Piepers et al., [5]. The corresponding secondary mAb for the detection of all immune somatic cells, macrophages and PMNs were PerCP-labeled anti-IgG2a mAb (R&D Systems, Minneapolis, United States), APC-labeled anti-IgG1 mAb (BD Biosciences) and FITC-labeled anti-IgM mAb (BD Biosciences, Becton Dickinson, France), respectively. In our study, the lymphocyte populations were mainly identified according to their size and granularity properties on the FSC/SSC dot plot. Epithelial cells were not detected in this flow cytometry gating scheme due to the exclusion of CD45 positive populations according to the product technical description (VMRD Inc.).

Three primary anti-bovine mouse mAbs were added in the 200 μL aliquots of cell suspensions standardized to 10^6^ cells/mL to a final concentration of 15 μg of each mAb /mL, according to the manufacturer’s labeling procedure (VMRD Inc.). After 30 min incubation with primary mAbs, the cells were centrifuged at 400×*g* for 5 min and washed twice with 200 μL of PBS. Subsequently, three secondary anti-mouse mAb were incubated to a final concentration of 10 μg of each mAb /mL. After 15 min incubation, two wash steps with 200 μL of cold PBS were performed using centrifugation at 400×*g* for 5 min. The cell pellets incubated with double mAb labeling were thus obtained.

Incubation with three isotype control antibodies was used as a control for the determination of non-specific background fluorescence in cytometry dot plots. Isotype control antibodies IgG2a (R&D systems), IgG1 and IgM (BD Biosciences) corresponding to CD45, C14 and CH138A, respectively (Table 1) were incubated in place of the primary mAb in identical blood and milk cell suspensions, and then incubated with the secondary mAbs as described above.

#### Incubation with viability kit for monitoring cell survival

To determine the viability of somatic cells and their subpopulations, the live/dead fixable violet dead cell stain kit (Invitrogen, Saint Aubin, France) was used prior to formaldehyde fixation and permeabilization. Live cells reacted with the fluorescent reactive dye active on extracellular and intracellular amines after transfer through cell membranes with damaged integrity. Therefore, the cell surface gives weakly fluorescent cells while cells with compromised membranes (considered as non-viable cells in our experiments) reacted with the dye throughout their entire cell volume leading to a brighter staining of cells. In both cases, the excess reactive dye was washed away after incubation period.

The cell pellets after labeling with primary and secondary mAbs were subsequently incubated with 1 μL reactive dye. Two hundreds μL of cold PBS was added after the incubation 30 min. The cell suspensions were centrifuged at 400×*g* for 5 min, and then washed twice. The final fixation step was performed by adding 200 μL of PBS containing formaldehyde 3.7% (v/v) to cell pellets. The suspension was fixed for a minimum of 15 minutes at 4°C, and according to the manufacturer’s procedure (Invitrogen), this fixed and labelled cell suspension could be stored for at least 3 months. In our experiment, all labeled cell suspensions were stored at 4°C for 5 days before flow cytometric analyses. Prior to the flow cytometry analyses, the stained cell suspensions were centrifuged at 400×*g* for 5 min, washed once and finally conserved in PBS containing 1% BSA (w/v) (Sigma-Aldrich, Saint-Quentin Fallavier, France).

### Microscopic Analyses

Optical microscopy was used in parallel of flow cytometry to measure the viability of the cell suspension under different physico-chemical treatments. The cell suspensions from the same milk cell pool were incubated with another live/dead kit, the BacLight™ bacterial viability kit, (Invitrogen). All steps were carried out in the dark at 4°C. The 200 μL cell suspensions at 10^6^ cells/mL were diluted 10 times with cold PBS. Cells were stained with the live/dead kit containing syto 9 and propidium iodide fluorescence markers that were diluted to a final concentration of 5% (v/v). Stained cell suspensions were immediately observed by the BX51 system microscope (Olympus, Rungis, France) equipped with two fluorescence channels, a dual band green/red channel (excitation wavelength 480-510/560-580 nm, emission wavelength 520-550/600-650 nm) and a single red fluorescence channel (excitation wavelength 560–580 nm, emission wavelength 600–650 nm). The objective ×10 was chosen to provide sufficient numbers of cells for analysis in a minimum number of viewed fields (n = 9). For each field, two images observed with the green/red dual band channel and the single band red channel were taken to measure the total and dead cell numbers. Archimed and Histolab software (Microvision instruments, Corbeil-Essonne, France) were used to count the cell numbers. Filter criterion (particle diameter > 4 μm) was applied to eliminate bacteria, cell debris and other undesirable particles. The cell viability was calculated by the following formula:

$$Viability\;(\%)=\frac{\mathrm{Number}\;(\mathrm{total somatic cells})-\mathrm{Number}\;(\mathrm{Dead somatic cells})}{\mathrm{Number}\;(\mathrm{total somatic cells})}\times 100\%$$

### Physico-chemical treatments applied to collected somatic cells from milk

Various physico-chemical treatments were applied to the collected somatic cells standardized to 10^6^ cells /mL, to reflects some kinds of stresses they have to face during milk technological processes: (i) the cell suspensions were heat-treated for 30 min at 39°C, 60°C or 80°C in a water bath in which the cells in the Eppendorf reached the targeted temperature in 3min for the conditions used; and cell suspensions heat-treated at 80°C were also used as the control to obtain the minimum value of milk cell viability; (ii) the cell suspensions were treated with various centrifugation rates, 400×*g*, 1500×*g*, 3000×*g*, 5000×*g* for 10 min at 4°C; (iii) the storage of the cells in milk *vs* sterile PBS was evaluated on a three-day test at 4°C. For this latter test, raw milk was stored for 24, 48 and 72h at 4°C before the cells were collected by centrifugation, washed in PBS and prepared under the same conditions as previously described for flow cytometry and microscopy analysis. In parallel, aliquots of cells collected the first day were kept stored in PBS during this same time period.

### Statistical Analyses

Flow cytometry data and microscopy data were statistically analyzed with StatBox^®^ version 6.3 (Grimmer logiciels, Paris, France). Differences were considered statistically significant at *P*<0.05. When the data concerned only the total cell viability, determined by flow cytometry and microscopy, the data were analyzed using one-way analysis of variance (ANOVA) When the data concerned the cell viability of the different subpopulations observed after the various physico-chemical treatments we used a two-way ANOVA in order to assess the main effect of each independent variable (each cell subpopulation, each physiochemical treatment) but also if there is any interaction between them. Both one-and two-way ANOVA were followed by the Newman Keuls test at the 95% confidence level.

## Results

### Viability of differential somatic cell counts by flow cytometry

**Fig 2:**
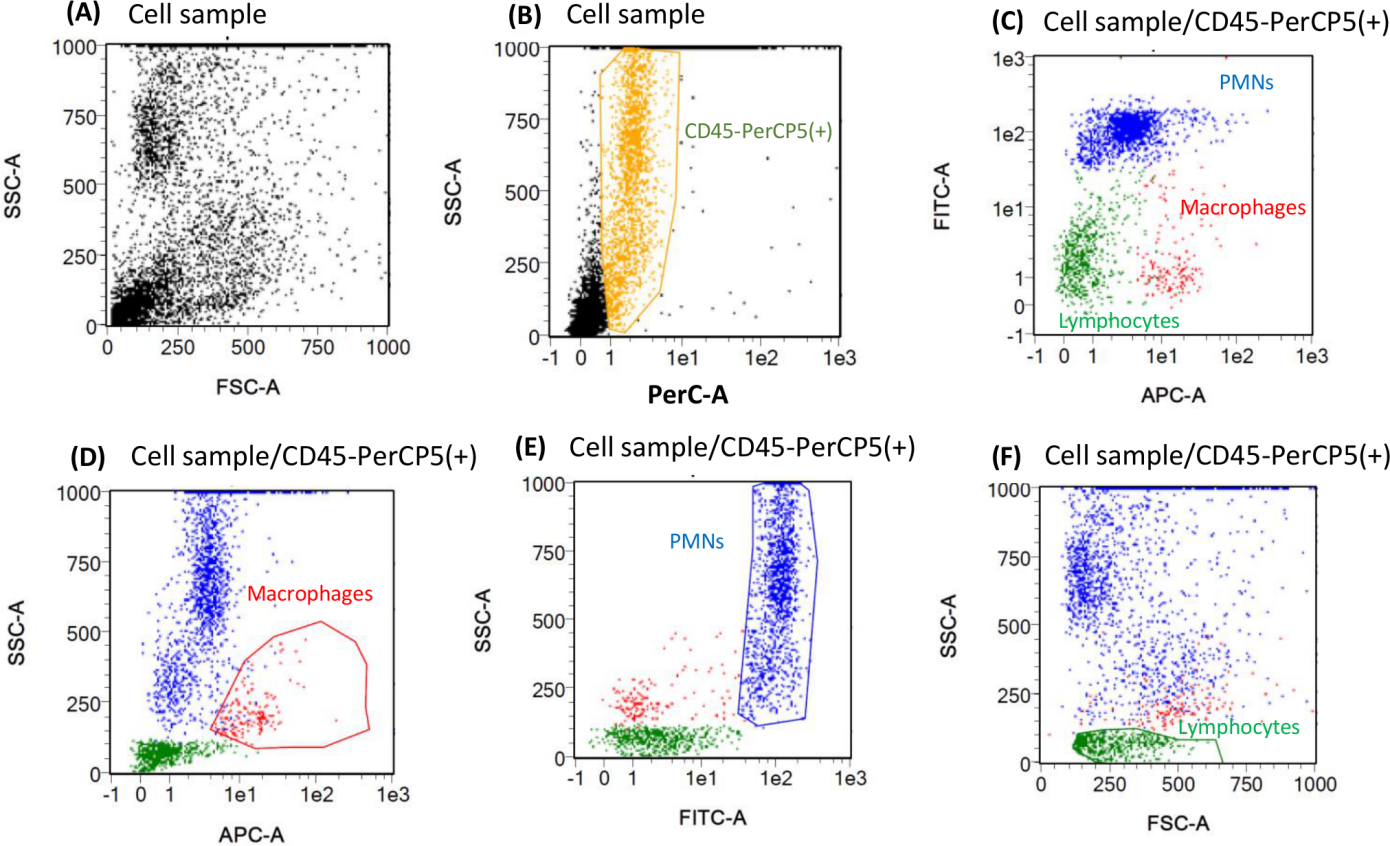
Flow cytometry scatter pattern for the identification of differential somatic cells in blood-milk mix cell suspension (1/1, v/v) and corresponding isotype control sample, respectively. (A) FSC/SSC dotplot of cell suspension. All somatic cells (in yellow) in cell suspension (B) were identified by CD45/PerCp+. The subpopulation of cell suspension in APC/FITC dotplot (C), macrophages (red), PMNs (blue) and lymphocytes (green) are identified by CD14/APC+ gate in APC/SSC plot (D), CH138A/FITC+ gate in FITC/SSC plot (E), and FSC/SSC size/granularity gate (F), respectively.

The different types of somatic cells were firstly identified prior to measuring the cell viability. Fig 2 shows the flow cytometry gating strategy that we developed by mixing cells collected from fresh blood and from raw milk.

All the particles present in the cell samples were firstly analyzed in the FSC/SSC dot plot (Fig 2A). Three main regions were gated in low FSC/low SSC, low FSC/high SSC and middle FSC/middle SSC regions of the plot. After labeling with specific primary mAb CD45 and with secondary mAb PerCP (written as CD45/PerCP), somatic cells were positioned as CD45/PerCP positive population in PerCp/SSC dot plot (Fig 2B). All cell CD45/PerCp+ population in PerCP/SSC dot plot were illustrated as APC/FITC dot plot, and the three cell types were simultaneously displayed in Fig 2C as monocytes in blood and as matured cells called macrophages in milk (in red), PMNs (in blue) and lymphocytes (in green) were separately found as APC+/FITC- and APC-/FITC+, APC-/FITC-, cell populations, respectively. They were also shown in three separated SSC scatter plots: (i) macrophages as CD14/APC+ population in APC/SSC dot plot (Fig 2D); (ii) PMNs as CH138A/FITC+ population in FITC/SSC dot plot (Fig 2E). Non-specific binding did not significantly affect fluorescence signal; (iii) lymphocyte as low SSC cell population in FSC/SSC dot plot according to their size and granularity properties (Fig 2F) in agreement with Mehne et al [7].

**Fig 3:**
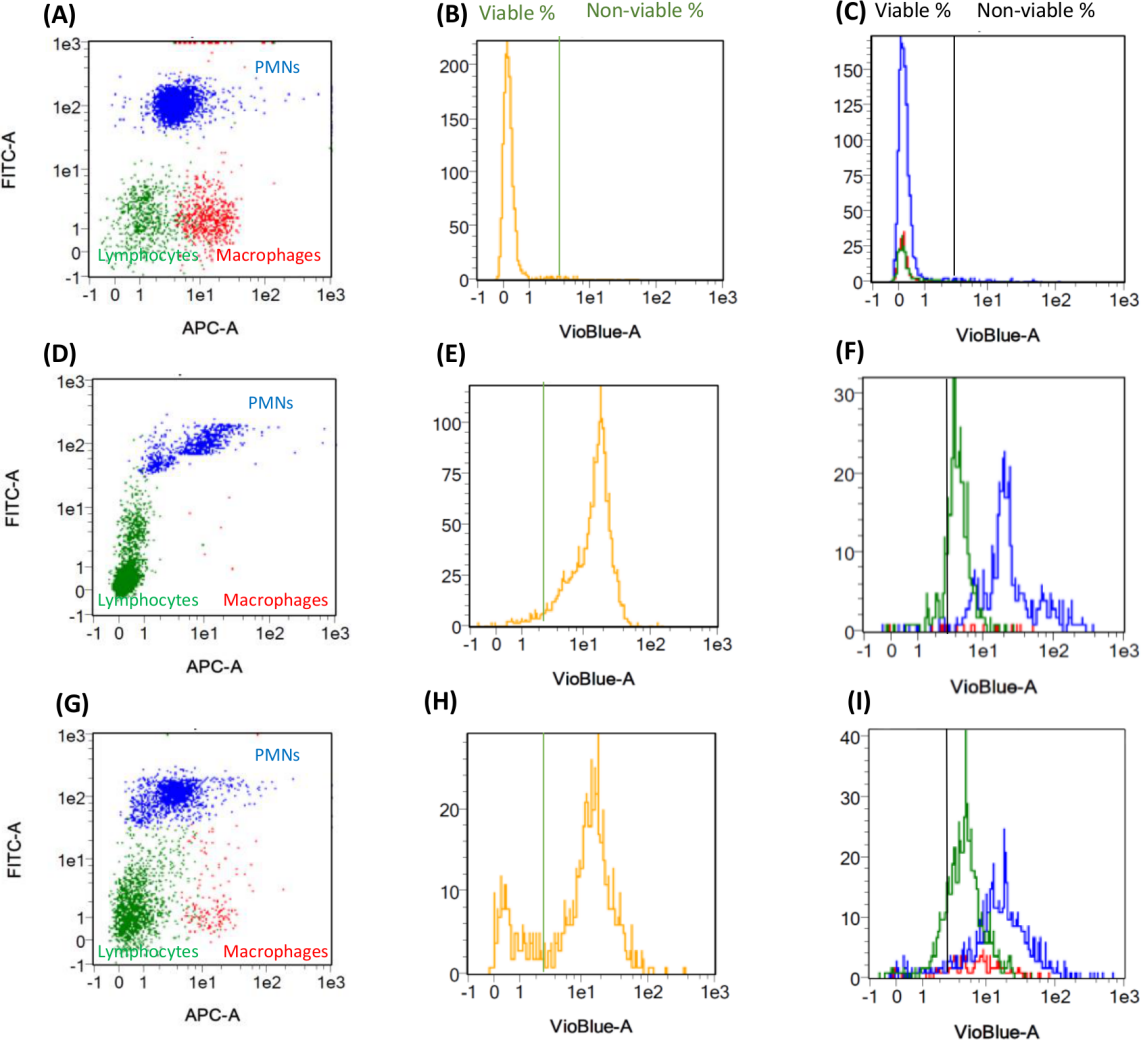
Flow cytometry identification of differential somatic cell count and simultaneous their quantification of all cells and each cell type. (A)(B)(C) fresh blood cell suspension, (D)(E)(F) milk cell suspension after 80°C × 30 min and (G)(H)(I)milk cell suspension conserved at 4°C in PBS pH 7.4. The cell subpopulations of each sample were shown in APC/FITC dotplot (A)(D)(G), the viable and non-viable population of all cells and each subpopulation were shown in histogram-Vioblue scatter (B)(E)(H) and (C)(F)(I), respectively.

To determine the cell viability, cell population was considered either as a whole or at subpopulation level. The viability of all cells was quantified by the vioblue/SSC plot (Fig 3).

The peaks on the left and on the right representing the viable and non-viable cell populations respectively were clearly separated by a threshold line. The position of this line was determined using both control samples: fresh blood cell suspensions as the live cell control sample and the milk cell suspensions after 30 min at 80°C as the dead cell control sample. The maximum value of cell viability was observed by the vioblue/FITC dot plot 95.9% for all cells in blood samples (Fig 3B). From the APC/FITC dot plot (Fig 3A) giving the types of cells present, was estimated the viability of the blood cell subpopulations (Fig 3C) which was 96.4%, 96.5% and 88.6% for monocytes, PMNs and lymphocytes, respectively. The minimum viability values were 0.7% for all cells in heat-treated milk sample (Fig 3E), and 6.3%, 0.4% and 2.8% for macrophages, PMNs and lymphocytes, respectively (Fig 3F). Additionally, the fresh milk cell suspensions that was stored at 4°C in PBS pH 7.4 (Fig 3G to 3I) showed an average population distribution of macrophages, PMNs and lymphocytes of 16.2%, 50.1% and 33.7% respectively (Fig 3G). The viability of all milk somatic cells was 39.5% for all cells (Fig 3H), and for each subpopulation the viability was 32.6%, 26.7% and 58.3% for macrophages, PMN and lymphocytes respectively (Fig 3I).

### How the cells resisted to various physico-chemical treatments

When the cells were submitted to heat, various centrifugation rate and storage duration at 4°C in order to mimic physico-chemical treatments that can be encountered during milk processing, the cells resuspended in PBS at 4°C were considered as the reference cell samples.

#### Heat Treatments

**Fig 4:**
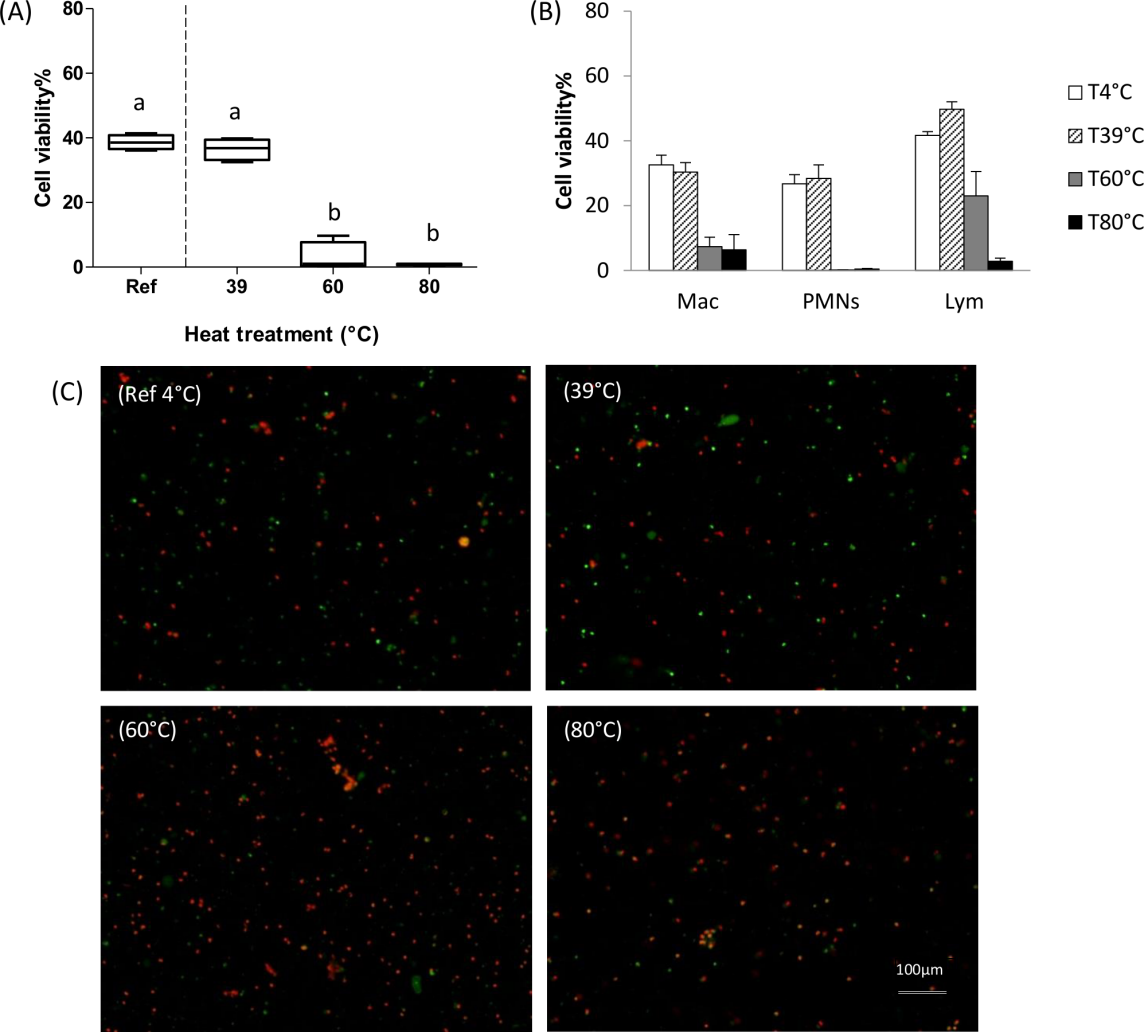
Cell viability of reference cell suspension (4°C) and of identical cell suspension with different heat treatments (39°C, 60°C, 80°C during 30 min). (A) Boxplot and whiskers of flow cytometry results with milk somatic cell suspensions incubated with vioblue Live/Dead kit (n = 4). (B) Cell viability of each type cell for 4°C (white bars), 39°C (hatched bars), 60°C (grey bars), 80°C (dark bars) centrifugations by flow cytometry; (C) Microscopy images (objective ×10) of milk somatic cell suspensions incubated with syto 9 and propidium iodide Live/Dead kit (n = 9). Means with different superscripts (a and b) differ significantly (*P*<0.001).

Various heat treatments (39, 60, 80°C for 30 min) were applied to the standardized cell suspensions. Fig 4A and 4B show the viability of all cells and of each cell type determined by flow cytometry respectively. The cell viability at 39°C was 39.5%, similar to that of the reference cell samples at 4°C, while the cell viability decreased dramatically down to 3.0%, at 60°C, as for the dead control cell sample (at 80°C) at 0.7% (*P*>0.05).

The same tendency was confirmed by microscopy (Fig 4C) (*P*<0.01) even if the cell viability values were slightly higher (16%) with this method. A brighter fluorescence intensity of syto 9 was observed for the cell suspension treated at 39°C compared to the reference sample at 4°C despite their similar viability values. For cell suspensions treated at 60°C and 80°C, 18% of all particles, were still stained with syto 9 fluorescence as live cells. Using flow cytometry and microscopy methods, the cell suspension exhibited a thermal resistance to the 39°C × 30 min condition but not to the 60°C × 30 min condition.

#### Various Centrifugation Rates

**Fig 5:**
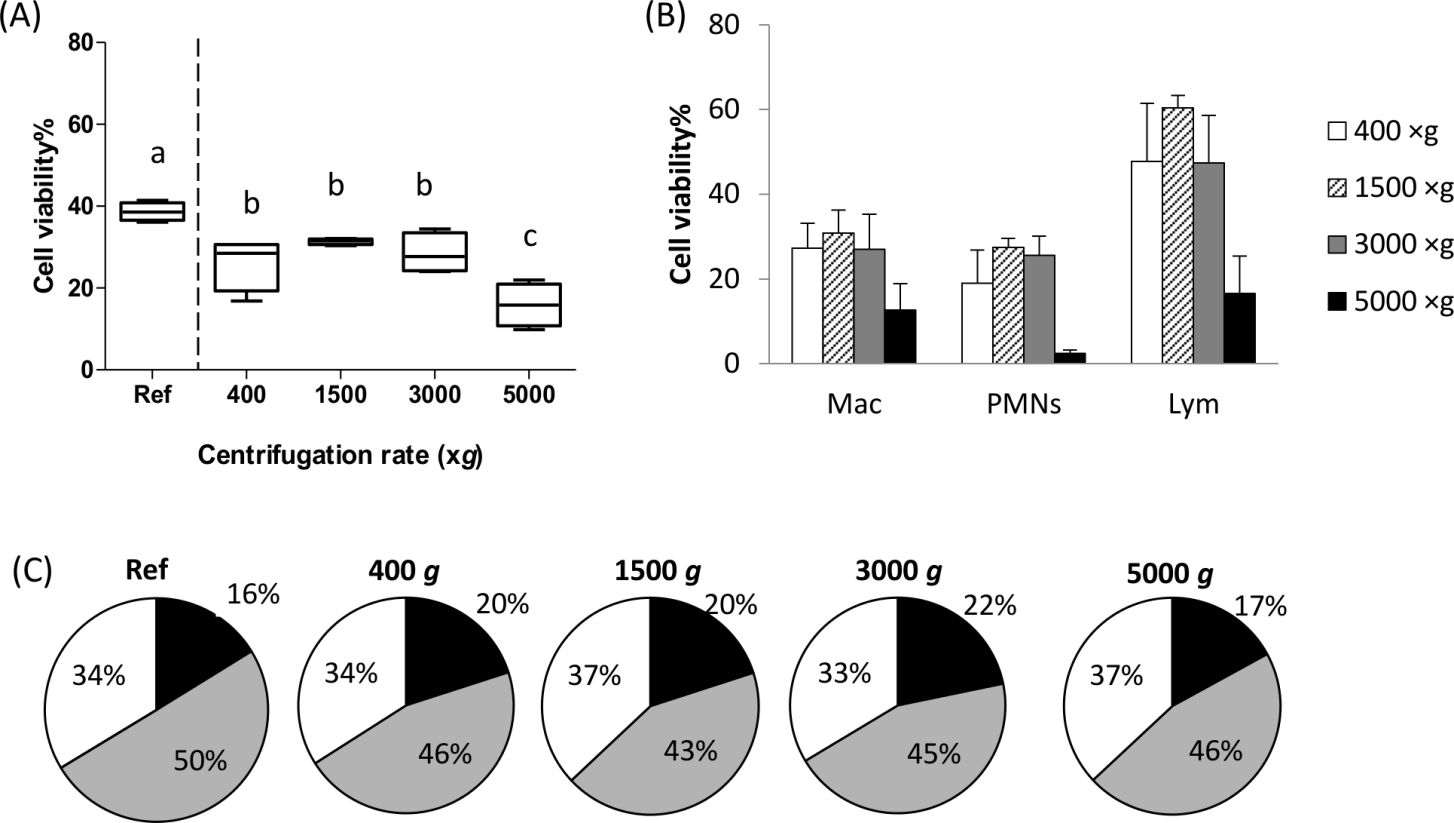
Cell viability with various centrifugation rates (400, 1500, 3000, 5000×*g* during 10 min at 4°C) comparing to the reference milk cell suspension without supplementary centrifugation (Ref). (A) Boxplot and whiskers of flow cytometry results with milk somatic cell suspensions incubated with specific antibodies and vioblue live/dead kit (n = 4); (B) Cell viability of each type cell for 400×*g* (white bars), 1500×*g* (hatched bars), 3000×*g* (grey bars), 5000×*g* (dark bars) centrifugations by flow cytometry; (C) Mean proportion of macrophages (dark sectors), PMNs (grey sectors) and lymphocytes (white sectors) in cell samples under various centrifugation with different gravitational velocities. Means with different superscripts (a-d) differ significantly (*P*<0.05).

The centrifugations at 400, 1500, 3000, 5000×*g* for 10 min at 4°C were applied to the cell suspensions of the same milk cell pool. Fig 5 illustrates the cell viability of the cell suspensions after recovering cells at various centrifugation rates measured by flow cytometry.

Flow cytometry results regarding the cell viability of all cells are illustrated in Fig 5A. Compared to the reference milk cell sample, the cell viability obtained by flow cytometry decreased from 39.5% to 29.7% for the cell suspensions treated at 400, 1500 and 3000×*g*, and to 9.4% for cell suspensions treated at 5000×*g* (*P*<0.01). For each cell type, their viability and their distribution in cell suspensions measured by flow cytometry were given in Fig 5B and 5C. The macrophages, PMNs and lymphocytes reacted differently in terms of cell viability under different centrifugation treatments (*P*<0.01), whereas their distribution in cell suspension kept the similar levels at 19.7%, 44.9% and 36.9%, respectively. Actually, the level of viable macrophages remained stable at 27.1% for the 3000×*g* treatment but decreased to 12.7% for the 5000×*g*. The viability of PMNs and lymphocytes was maximal at 1500×*g* and minimal at 5000×*g* respectively.

#### Storage Conditions

The flow cytometry results of the cell distribution and the viability under different storage conditions (in PBS and milk for 24, 48 and 72h) were shown in Table 2.

**Table 2:** Flow cytometry results concerning the proportion of cell subpopulation and their viabilities of milk cell suspensions in PBS and milk conservation during 24, 48, 72h (n = 4).

Storage media and duration		Proportion of subpopulation (%)			Cell viability (%)			
		Macs	PMNs	Lyms	All cells	Macs	PMNs	Lyms
Reference sample (6h) *		16.2±1.6 ^d^	50.1±1.7 ^b^	33.7±3.1 ^c^	39.5±1.2 ^A^	32.6±3.0 ^bc^	26.7±2.7 ^c^	58.3±1.1 ^abc^
	24 h	17.5±2.4 ^d^	48.3±3.5 ^bc^	34.2±5.6 ^bc^	35.7±5.7 ^B^	46.6±5.7 ^abc^	15.6±0.7 ^bc^	55.0±3.9 ^ab^
Conservation in PBS	48 h	5.8±0.5 ^d^	77.9±1.7 ^a^	16.4±1.2 ^d^	29.4±1.9 ^B^	37.0±6.4 ^abc^	14.5±0.8 ^d^	47.0±1.9 ^abc^
	72 h	7.5±0.9 ^d^	76.3±4.8 ^a^	16.2±2.7 ^d^	16.4±1.9 ^C^	31.2±2.4 ^bc^	4.9±1.9 ^d^	11.4±3.1 ^d^
Reference sample (6h) *		16.2±1.6 ^de^	50.1±1.7 ^b^	33.7±3.1 ^bcd^	39.5±1.2 ^A^	32.6±3.0 ^bcde^	26.7±2.7 ^cde^	58.3±1.1 ^abc^
	24 h	18.2±2.4 ^cde^	47.3±3.4 ^b^	34.4±6.8 ^bc^	31.6±1.4 ^B^	37.0±2.4 ^bc^	32.5±3.4 ^bcd^	29.9±1.6 ^cd^
Conservation in Milk	48 h	8.6±2.3 ^e^	72.8±1.3 ^a^	18.6±1.1 ^cde^	36.7±2.8 ^A^	30.8±6.1 ^bcde^	35.8±3.1 ^bcd^	20.6±4.5 ^de^
	72 h	8.1±0.5 ^e^	78.3±8.2 ^a^	13.6±0.6 ^de^	37.8±4.3 ^A^	41.6±5.7 ^abc^	34.0±2.9 ^bcde^	25.3±3.0 ^cde^

*Reference sample was the milk cell suspension obtained 6h after the collection of fresh raw milk and kept in PBS at 4°C. These reference values were compared to both PBS and milk storage conditions. They were cited twice here because of the statistical group. The abbreviation of macrophages, polymorphonuclear neutrophils and lymphocytes were written as Macs, PMNs and Lyms, respectively. For the proportion of subpopulation and cell viability, means with different superscripts (a-e) in each trial differ significantly (*P*<0.05), means with different superscripts in capital letters (A-C) differ significantly (*P*<0.05) and NS-no significant difference (*P*>0.05).

Compared to the reference milk cell sample, the cell distributions after 24h were not significantly modified (*P*<0.05) either stored in PBS or in milk. Macrophages, PMNs and lymphocytes stored in both PBS and milk samples were present on average at 17.8%, 47.8% and 34.3%, respectively. After 48h and 72h of storage, the percentages of macrophages and lymphocytes decreased in both PBS and milk, and their average distribution decreased down to 7.8% and 14.9%, respectively; while PMNs became predominant, over 75% after 72h storage, in milk or in PBS, in contrast to the behavior of the macrophages and lymphocytes.

Concerning the cell viability, a better cell conservation was observed in milk than in PBS, in particular after 72h of storage. For all cells, a decrease in cell viability in PBS was estimated from the original value of 39.5% to 35.7% (24h), to 29.4% (48h) on average, and down to 16.4% after 72h storage. In contrast, cell viability remained stable with 37.8% viability found after 72h incubation in milk for all the cells. Throughout the storage time, macrophages remained viable in both PBS and milk; PMN viability decreased from 26.7% to 4.9% in PBS but was noted to be very stable in milk samples; lymphocyte viability decreased significantly from 58.3% to 11.4% in PBS and to 25.3% in milk.

## Discussion

We developed a new flow cytometry approach to quantify the cell viability of each type of milk somatic cells present in milk and their resistance to various physico-chemical treatments. Even few studies took into account the viability of these somatic cells [5,8,16], to our knowledge, none provided information on their live-or-dead status under different physico-chemical conditions or during milk processing.

We found similar trends of cell viability by using both flow cytometric and conventional microscopy, but flow cytometry provides as expected supplementary information regarding cell distribution and cell viability at the subpopulation level (Fig 4). Furthermore, the live/dead fixation kit used in our experiment made flow cytometry a better approach with (i) longer preservation of fixed samples up to months according to the manufacturer’s procedure; (ii) great choices of multiple multicolor fluorescence markers such as red, green, blue and violet that do not cross-react and limit the degree of compensation required. Nevertheless, higher absolute values of viability were obtained with microscopy results. The discrepancies between the results obtained by the flow cytometer and the microscopic analyses could be partly explained by the exclusion of epithelial cells by flow cytometry, the bias of microscopic software, or the false-positive staining of cellular debris or other impurity produced during different treatments with fluorescence syto 9 (data not shown) counted from microscopic observation.

We used the graphic layout of FSC x SSC, size and granularity, although the layout of cells by size and granularity is not as defined in milk as in samples blood. Such a choice was a compromise between the number of antibodies used and the analysis of the sample by flow cytometry during an appropriate scale of time. It would be possible to complete the identification of lymphocyte at the population and sub-populations by using antibodies directed against T lymphocytes (CD4 and CD8) and B cell for example [17–24]. A as matter of antibody choice, the use of CD14 was based on the fact that these cellular receptors are expressed by monocytes, macrophages and polymorphonuclear cells [25,26]. CD14 is involved in a number of biological and immune responses and when expressed in leukocytes and functions as a key molecule in the recognition of invading pathogens and trigger the cascade of inflammatory reactions [27]. Some authors have reported the importance of CD14 expression by neutrophils [27–29]. Even Burton and Erskine [28] emphasized that most of bovine neutrophils do not express surface CD14 molecules but they have stored in cytoplasmic granules ready for action. When blood neutrophils migrate, the granules containing CD14 to follow the surface of the cells where those molecules can interact with components of the bacterial wall. This theory cited by Burton and Ersksine [28] can be recently confirmed by Sladek and Rysanek [16].

We paid great attention to have appropriate control samples, to fix the range of maximum and minimum cell viability values. We chose cow blood and heat-treated milk samples to ascertain the limit values of viability, which were 95.6% for blood and 0.7% for heat-treated milk cell samples, in agreement with literature data [5,30]. For example, Paape et al. [30] showed an elevated viability of PMNs from blood and milk at 97% and 95% respectively. Piepers et al. [5] demonstrated that PMN viability values ranged widely between 25.7–92.8% in bovine milk. Nevertheless, it is still difficult to obtain all cells considered as totally dead cells with the same cell properties as viable cells because of various cell morphology properties of apoptotic and necrotic cells.

Our study provided, for the first time, cell viability values for each cell type in bovine milk and under various physico-chemical conditions that can be encountered during dairy technological processes. Thus, it gives possibility to explain the cell behavior in dairy products processes. With regard to the heat treatment, milk somatic cells kept the same viability between 39°C, which corresponded to the corporal temperature of healthy cows ranging from 38.5 to 39.2°C [31] and 4°C, the temperature currently used for milk storage. In contrast, a drastic decrease in cell viability to 3.0% and 0.7% was observed at 60°C and 80°C, respectively, conditions that can be encountered during high temperature treatments such as pasteurization (63°C, 30 min) and ultra-high temperature UHT (>140°C, 2–3 seconds) in dairy products [32]. We also observed with the microscopic images that the cells even dead maintain their overall shape, suggesting that they kept inside the cells most of the enzymes that can be further released into the dairy products.

We did not observe great changes in the distribution of each cell types when centrifugation rate was increased up to 5000 *g* ×10 min, and this fact gives rise to multiple ways to collect cells from milk without impacting the overall population in an appropriate and defined range of centrifugation rate. Currently, the centrifugation rate generally used to obtain the cell pellet, has a large range from 200×*g* to 42000×*g*. [3,33,34] and this brings up question about the viable status of the somatic cells at higher rates even if the total number of collected cells is certainly higher than at lower rate. The centrifugation steps used in our study are compatible with those used during technological processes when milk is skimmed or bactofugated.

We showed that somatic cells were well preserved for 72 h in milk, which is close to blood in terms of osmotic pressure, pH and nutrient supply, than in PBS, confirming that milk can be used as a good conservation medium [35]. We cannot preclude that bacteria that are present in raw milk as natural contaminating flora may have influence on the viability of somatic cells during storage, but this was not explored in this study. According to the low level of bacteria initially present in raw milk, we can suppose that no dramatic growth would have been occurred at 4°C during storage in agreement with FAO estimation of the multiplication factor (5.5 fold) of aerobic mesophilic flora at 4.5°C [36].

As an important source of enzymes such as lipases and proteinases, milk somatic cells may influence the initial quality of milk and final quality of dairy products through their enzymatic activities [12]. The viability of somatic cells would be a key criterion to study the liberation of their endogenous enzymes in the dairy matrix and therefore to complete the fingerprint of somatic cells in milk, from the distribution of cells to the viability and the capability of enzyme release [12]. Thus, the differentiation of somatic cell subpopulations and the quantification of their viability by this cytometry approach will be advantageous to study the accessibility of endogenous enzymes from each cell type in milk. If intact viable somatic cells could be considered as an enzymatic reservoir, the beneficial use of these enzymes (e.g. collect these somatic cells from milk and then use them in dairy field) can be a possible way of improving the dairy processing and the quality of the final products, apart from a mastitis context. The fact that the somatic cells remain largely viable in milk after 72h provided a delay for milk collection with live somatic cells. Using this cytometric approach, the distribution and viability of somatic cells at a subpopulation level was detected under different treatments, which can provide valuable arguments to explain their action in a more complex dairy matrix.

## Conclusion

The present study showed a new flow cytometry approach for simultaneously identifying somatic cells and quantifying their viability at a subpopulation cell level in bovine milk. This novel cytometric approach provides a new array to better understand milk cell biology and to further establish the relationship between the cell viability and the release of these endogenous enzymes in dairy matrix.
